# VSpipe, an Integrated Resource for Virtual Screening and Hit Selection: Applications to Protein Tyrosine Phospahatase Inhibition

**DOI:** 10.3390/molecules23020353

**Published:** 2018-02-07

**Authors:** Sandra Álvarez-Carretero, Niki Pavlopoulou, James Adams, Jane Gilsenan, Lydia Tabernero

**Affiliations:** 1 School of Biological Sciences, Faculty of Biology Medicine and Health, University of Manchester, Manchester Academic Health Science Centre, Manchester M13 9PT, UK; sandra.ac93@gmail.com (S.Á.-C.); nikipavlopoulou@gmail.com (N.P.); james.adams-6@postgrad.manchester.ac.uk (J.A.); Jane.Gilsenan@manchester.ac.uk (J.G.); 2 School of Biological and Chemical Sciences, Queen Mary University of London, London E1 4NS, UK; 3 Insight Centre for Data Analytics, NUIG, Galway H91, Ireland

**Keywords:** computational screening, virtual screening (VS), protein tyrosine phosphatase (PTP), ligand efficiency indices (LEIs), drug discovery

## Abstract

The use of computational tools for virtual screening provides a cost-efficient approach to select starting points for drug development. We have developed VSpipe, a user-friendly semi-automated pipeline for structure-based virtual screening. VSpipe uses the existing tools AutoDock and OpenBabel together with software developed in-house, to create an end-to-end virtual screening workflow ranging from the preparation of receptor and ligands to the visualisation of results. VSpipe is efficient and flexible, allowing the users to make choices at different steps, and it is amenable to use in both local and cluster mode. We have validated VSpipe using the human protein tyrosine phosphatase PTP1B as a case study. Using a combination of blind and targeted docking VSpipe identified both new and known functional ligand binding sites. Assessment of different binding clusters using the ligand efficiency plots created by VSpipe, defined a drug-like chemical space for development of PTP1B inhibitors with potential applications to other PTPs. In this study, we show that VSpipe can be deployed to identify and compare different modes of inhibition thus guiding the selection of initial hits for drug discovery.

## 1. Introduction

The success of a drug discovery programme involves the optimisation of a large number of parameters that combine both target properties (biological space) and ligand properties (chemical space). Achieving the right balance of potency and pharmacological properties is what determines the success of a drug candidate in the clinic. However, this process is long and complex, thus speeding up the early stages of hit identification and lead selection are critical. High-throughput screening technologies are now common and they can generate vast amounts of information, however, experimental based screens are expensive and require access to large collections of compounds. Computational screening or virtual screening (VS) is relatively cheap and in principle there is no limitation on the chemical space to search. Thus, VS offers advantages at the early stage of drug discovery to identify compounds with potential inhibitory activity towards the target of interest [1,2]. Independent from the source of information (experimental or VS), the selection of starting chemical points for validation and subsequent optimisation remains challenging.

PTPs are particularly difficult drug targets because of their highly polar and conserved active site, thus most active site inhibitors are either not selective enough or exhibit poor cell permeability due to their charged nature [3,4]. PTPs play important roles in diseases [5,6] and, although they have been subjects of numerous drug discovery studies [3,7], no drugs are yet in the clinic for this class of enzymes. There are now 589 PTP structures available from different organisms (PDB release November 2017), which could be used in structure-based VS. This offers a clear opportunity to identify new potential inhibitors and new mechanisms of inhibition to target PTPs to fill the therapeutic gap.

Large-scale VS is still a time-consuming and error-prone method when done manually. It involves the preparation of the receptor protein and the compound libraries, ligand docking, and estimation of the binding affinity of each ligand according to a scoring function. This process sometimes requires the use of several software packages and the manipulation of a large number of files, a process that can be daunting for non-expert users. Even more overwhelming is the assessment and selection of initial leads from the output of a large-scale VS.

Generally, potency, inferred from binding affinity (experimental K_i_ or calculated ΔG), is the main criterion used [8,9], but high potency alone is not sufficient to guarantee the development of a good drug. Other physicochemical parameters impact on the pharmacokinetic properties of lead compounds and need to be taken into account to develop successful drug candidates, in particular orally bioavailable drugs.

Different approaches have been proposed to guide the medicinal chemistry efforts in order to mitigate the significant attrition rate during drug development, such as the “rule of five” [10] or the use of ligand efficiency (LE) (binding energy/non-hydrogen atom) [8,11]. An extension of the LE concept employs the binding efficiency index (BEI) and surface efficiency index (SEI) to guide the drug discovery process [12,13]. These ligand efficiency indices (LEIs) merge the chemical and biological space giving a read out on the potential druggability of the compounds. Therefore, LEIs serve as useful guideposts during the drug development process to exploit the most druggable chemical spaces for a given target [12].

We have developed a semi-automated, user-friendly virtual screening pipeline, VSpipe, that uses Autodock [14] and Autodock Vina [15] and that extracts results into a simple output file. A number of user-driven decisions allow predefining subsets of ligands to be docked, together with the opportunity to filter the results output according to physicochemical properties. We have also incorporated the visualisation of LEIs plots as part of the output, to aid in the identification and selection of hits for experimental validation. VSpipe offers a streamlined and integrated platform for VS that facilitates the identification and selection of suitable leads for experimental validation and drug development without requiring previous knowledge or expertise in drug discovery.

VSpipe was validated with the human protein tyrosine phosphatase PTP1B using a combination of blind docking and targeted docking with commercial libraries of compounds. VSpipe identified six clusters of ligand binders, including new and previously reported binding sites for substrates and high affinity inhibitors. Analysis of the LEI plots allowed an easy assessment of clusters according to their drug-like properties and the comparison between different modes of inhibition to identify a drug-like chemical space for this target. The same approach is applicable to other PTPs and may provide alternatives to the development of active site inhibitors.

## 2. Results

### 2.1. Overview of VSpipe

**Figure 1:**
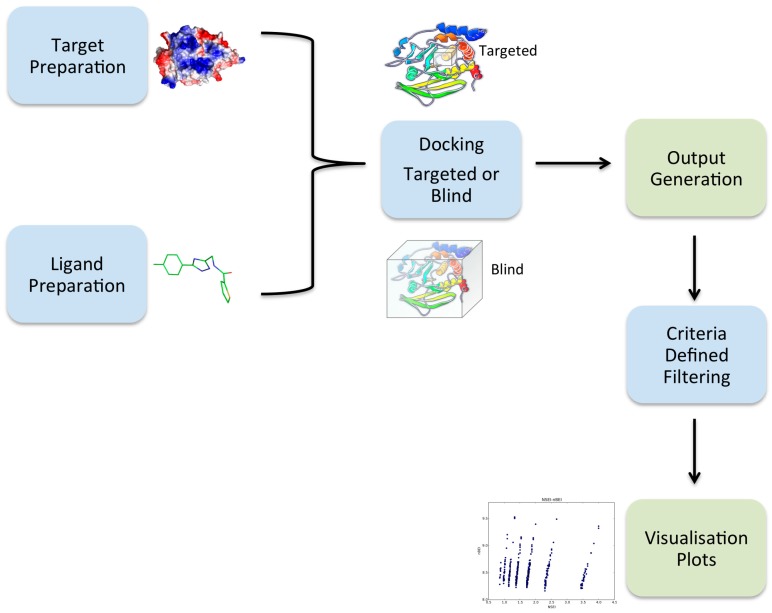
Overview of the VSpipe workflow. The diagram highlights the main steps that take place during VSpipe. The blue boxes show the tasks VSpipe performs after reading input information by the users, whilst the green boxes represent outputs ready for analysis.

VSpipe integrates a number of different tasks that are required to progress along a structure-based VS process as well as extracting results to produce visual outputs. The tasks include: (i) preparation of the receptor protein; (ii) preparation of the ligands and their conformers; (iii) docking of the ligands on the receptor; (iv) extraction of coordinates for the best binding poses and preparation of a results summary; (v) filtering of the docking output; and (vi) generation of visualisation plots ready for analysis (Figure 1).

A key objective during the development of VSpipe was to generate a tool that was flexible and yet user-friendly for the non-expert users. In order to achieve this, we decided that VSpipe should interact with the users during the VS process by outputting instructions, questions and tasks status on the terminal from the beginning to the end of the process. Therefore, the users follow step by step all the tasks a VS needs, they select the best option to process the data at each step, and they are informed about the status of the tasks being carried out by VSpipe. VSpipe aims to target a broad range of users, from those with a high level of understanding of VS to those who are new to the field. Therefore, we think this interactive design is easy to manage at any level of expertise and it may be useful for educational training as well.

We developed two modes of VSpipe, one to run on local personal computers (VSpipe-local) and another to run in a computer cluster (VSpipe-cluster). VSpipe-cluster provides higher performance when screening big compound libraries (>6000 compounds). Currently, this mode has been designed to run on the Data Processing Shared Facility (DPSF), a High Performance Computing (HPC) cluster at the University of Manchester (UoM), therefore, modifications will be required to perform in other platforms (see methods).

### 2.2. VSpipe-Local Mode

The VSpipe workflow depends on the mode used, local or cluster. When VSpipe-local is executed, the tasks are divided into two blocks: preparation and settings (*Block1*) and docking, results extraction and filtering (*Block2*). During *Block1*, the users are guided through different questions on their preferences about the preparation of the input files and the kind of docking to be performed. These choices are saved in a configuration file, which is later used in *Block2* (Supplementary Materials Figure S1). Preparation of the receptor protein (PDB file) involves the elimination of pre-existing ligands, water molecules, ions, and the selection of a single chain. Preparation of the ligands ensures that incomplete or repetitive records are eliminated, missing physicochemical properties are calculated, and that energy minimised atomic coordinates are generated for conformers prior to docking [16] (see Methods and Documentation).

In *Block1*, users can choose to use their own ligands or from a number of compound libraries (21 commercial libraries). There is also the option to apply Lipinski rules [10]. A detailed explanation about all the possible user decisions and steps performed in *Block1* when running VSpipe-local can be found in the Supplementary Figure S1.

In *Block2*, the docking is performed with AutoDock [14] (AD4) or AutoDock Vina [15] (Vina) according to users’ selection. Docking can be performed over the whole protein surface (blind docking) or around a defined region (targeted docking), depending on previous knowledge of the target. The results from the docking are summarised in a comma-separated file and in a tab-separated file and the lowest energy ligand poses extracted. The summary file includes 16 physicochemical and LEIs parameters for each compound (see methods for details).

If users have already performed a previous VS and want to change certain parameters to repeat the docking, then they proceed directly to *Block2*. In this case, the users can update or change their previous selections (Lipinski rules, docking tool, ligand library and mode) before starting the docking. The workflow of *Block2* is shown in the supplementary Figure S1. When virtual screening is finished, the user can choose to filter the results. The filtering script reads the comma-separated output file and offers the option to filter by individual chemical properties and LEIs, as well as to select a threshold value for the chosen parameter. This process can be done in an iterative manner to apply successive filters. In the end, a new results summary file is generated together with the visualisation plots (PSA, MW, logP, HBA, SEI/BEI and NSEI/nBEI), and the corresponding subset of ligand PDB files ready for analysis.

### 2.3. VSpipe-Cluster Mode

VSpipe-cluster was designed to use the UoM-DPSF capabilities efficiently while still taking into account user preferences, thus the workflow is different from the one in the local mode (Supplementary Materials Figure S2). The first step runs the bash script (*pipeline_executable*) that creates all the directories to be used during the VS and the variables file. Once all input files are uploaded, the user decides which components of VSpipe-cluster will be submitted depending on the type of docking to be used (targeted or blind), the type of software to be used (AD4 or Vina), and whether the receptor or ligands need to be prepared. Note that the path variables to the directories where the AutoDock and VSpipe-cluster reside in the DPSF are defined by default in these scripts. Therefore, if VSpipe-cluster is to be used in another HPC cluster, it will require changes in VSpipe’s main code to meet specific cluster requirements. Although the running of VSpipe-cluster is a bit more laborious, it is still fairly simple to use it by following the instructions in the documentation and the process is significantly faster, particularly when performing large-scale VS.

### 2.4. Docking Module Performance

The computation processing time usage (CPU) needed to perform the docking, with either AD4 or Vina, was benchmarked by using both the VSpipe-local and VSpipe-cluster modes (Table 1). The results show how the docking is faster when using the HPC cluster (using VSpipe-cluster), where the parallelised versions of AD4 and the multithreaded Vina are used, as opposed to a local computer (using VSpipe-local). We implemented in VSpipe-cluster the two parallelised versions of AD4 available, the Message Passing Interface version (MPI) and the Open Multiprocessing (OpenMP) and enabled the multithreaded version of Vina (OpenMP) so we could benchmark their performance. The OpenMP parallelism is based only on threads (i.e., the processes are split among the available threads as if they were for loops), whereas the MPI parallelism is based on both the processes and the threads (i.e., every process is independent from each other).

The OpenMP can be launched in a user-friendly way by just calling the program (easier to implement and debug in VSpipe-cluster). However, using the MPI version requires the users to check that all the programme dependencies have been properly set in the local environment and to specify the number of processes to be executed (more difficult to implement and debug in VSpipe-cluster). MPI versions can run in both shared and distributed memory architectures, while OpenMP versions can only run in shared memory architectures. Therefore, it is perhaps not surprising that the AD4-MPI version performed faster than the AD4-OpenMP (Table 1). Although the benchmarking was carried out with a small ligand library (98 compounds), we could observe an increase in the performance of the AD4-MPI when increasing from 8 to 60 core usage (from ~2 h to 9 min). However, the difference between using the Vina-OpenMP with 8 cores and with 16 cores was not significant (21 min versus 13 min). In fact, the larger the library used in the VS, the better the parallelisation performed. Therefore, even though the increase in the performance when using 8 or 16 cores was small with the 98 compounds, we have observed a better performance with Vina-OpenMP when using larger libraries (>6000 ligands). Consequently, VSpipe-cluster uses the AD4-MPI parallelised with 60 cores and the Vina-OpenMP parallelised version with 16 nodes.

Vina is the fastest docking option on both local and cluster modes. This is due to the gradient optimisation method, Iterated Local Search (ILS) global optimiser [17,18] within the local optimisation procedure algorithm Broyden-Fletcher-Goldfarb-Shanno (BFGS) [19] that it uses, which is faster than the Lamarckian Genetic Algorithm (LGA) [20] implemented in AD4. Therefore, we recommend using Vina for the initial screening or for those with large compound libraries.

### 2.5. Case Study on PTP1B

#### 2.5.1. Initial Pocket Identification

VSpipe includes two alternatives for the molecular docking, either blind docking or targeted docking around a defined site. Blind docking offers advantages when there is no previous knowledge of functional sites on the target protein. Blind docking was a feature we were keen to implement in the pipeline to explore if suitable binding sites could be identified when no presumptions are taken. When aiming for new pocket identification in a target, we recommend the use of blind docking with a small fragment library, as in our experience, this is sufficient to identify sites of interest and it is relatively quick (Supplementary Materials Figure S3).

Targeted computational screens using PTP proteins have been widely exploited to identify binding compounds at the active site [21,22]. However, the use of blind docking of compound libraries against PTPs to identify new druggable sites has not been reported before. To the best of our knowledge, blind docking on PTPs has only been reported to model the binding of an allosteric PTP1B inhibitor [23]. To explore the use of blind docking with VSpipe, we undertook a case study using the PTP1B structure (PDB ID:1T4J) and the Asinex fragment library (composed of 6243 chemical fragments) (see Methods for details).

**Figure 2:**
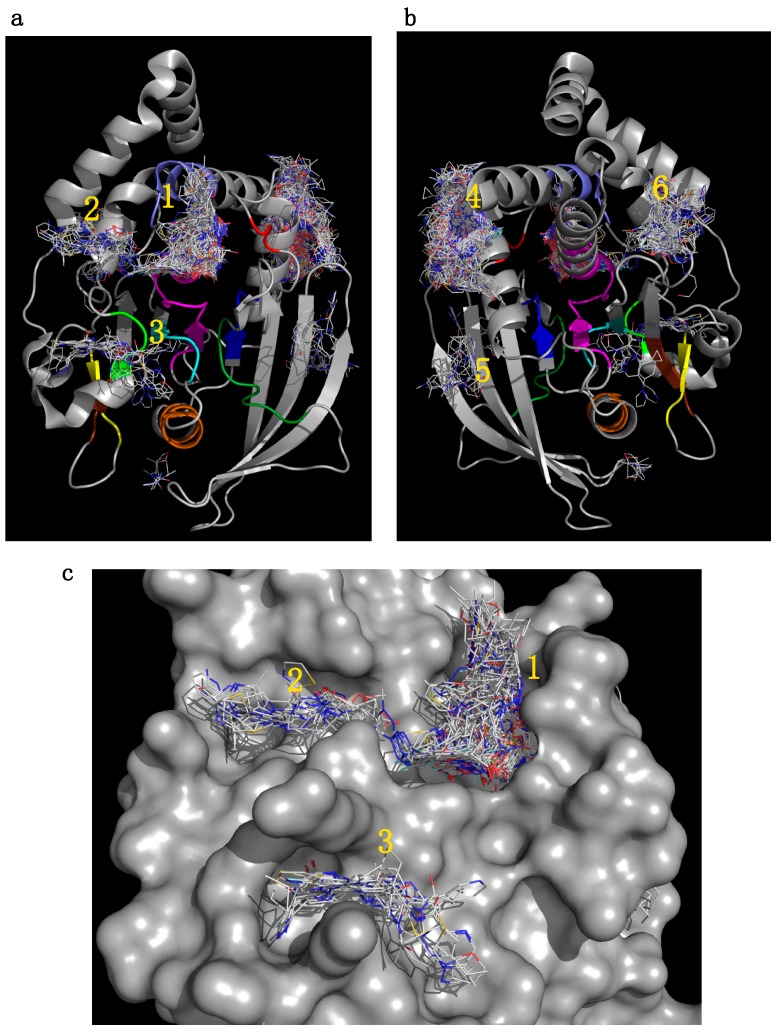
Blind docking results for PTP1B. The structure of PTP1B is shown as a grey cartoon, front view on (**a**) and back view on (**b**). Compounds are shown as lines. Areas where there is a high density of compounds (greater than 10) are classified as clusters and numbered: (1) active site, (2) secondary pTyr site, (3) centred on L41 (4) allosteric binding site (5) centred on V155 and (6) centred on E252. Key functional regions [24] have been coloured on the PTP1B structure, Motif 1 in green, Motif 2 in yellow, Motif 3 in brown, Motif 4 in cyan, Motif 5 in orange, Motif 6 in blue, Motif 7 in dark green, Motif 8 in red, Motif 9 in magenta and Motif 10 in purple; (**c**) Blind docking successfully identified the active site (1) and surrounding key sub-pockets including the secondary pTyr site (2) and the site around motif 1 (3). Compounds in the clusters are shown as lines whilst PTP1B shown as grey surface.

Blind docking identified six clusters of fragments (whereby a cluster was defined as greater than 10 fragments at a specific site) at different binding sites on the protein surface, including the active site (Figure 2). Additionally, we found four low-density binding sites with less than 10 fragments at each site, not classified as clusters according to our criteria. The same six clusters were observed regardless of the ligand library used (see methods). In fact, we could identify the four functionally relevant binding sites using a small 98-fragment library (Maybridge NCO) (Supplementary Materials Figure S3), suggesting that the identification of potential binding sites is independent from the library used and can be achieved quickly with blind docking and a limited number of ligands.

The positions of the six clusters were mapped on the structure of PTP1B (Figure 2) and included: (1) the active site [25], (2) the secondary phospho-tyrosine binding site [26], (3) a site centred on L41, (4) an allosteric binding site [27,28], (5) a site centred on V155, and (6) a site centred on E252. Importantly, all but two clusters (5 and 6), mapped to functionally important conserved PTP motifs [24,29] or to sites that have been previously identified in drug discovery [27,28,30,31], hence providing confidence in our approach. The additional clusters 5 and 6, mapped into new pockets not yet targeted for this enzyme, which could be explored in the future.

**Figure 3:**
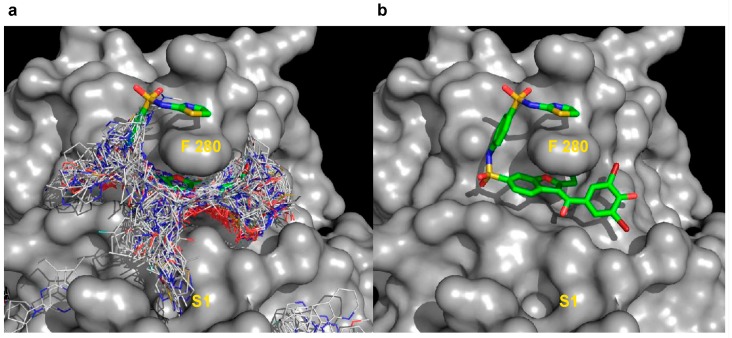
Compound cluster distribution at the allosteric site. Ligand cluster at the allosteric site. (4) partially overlaps with the binding site of BZ3 inhibitor reported in the literature [27]. (**a**) Overlay of the cluster at site 4 with compounds shown as lines on top of the allosteric inhibitor BZ3 (shown in green as sticks) and PTP1B as grey surface; (**b**) Crystal structure of BZ3 in complex with PTP1B. SAR during development of BZ3 was undertaken to optimise binding around F280 [27]. S1 represents a new sub-pocket identified in the screen with VSpipe that could be exploited to generate new allosteric inhibitors.

The cluster distribution at the allosteric site 4, maps with the binding of the allosteric inhibitor (3-(3,5-dibromo-4-hydroxybenzoyl)-2-ethyl-*N*-(4-(*N*-(thiazol-2-yl) sulfamoyl) phenyl) benzofuran-6-sulfonamide) referred from herein as BZ3, as shown by the crystal structure in complex with PTP1B [27] (Figure 3). Interestingly, cluster 4 shows a number of fragments distributing into a new sub-pocket S1 (Figure 3a) not occupied by BZ3 but nearby the benzofuran ring. Previous SAR investigations carried out by Wiesmann et al. [27] focused on extending binding around Phe280 to increase hydrophobic interactions (Figure 3b). Our results suggest that S1 may be a new area for future SAR investigations, adding interactions at this sub-pocket. These results indicate that using VSpipe to perform a blind docking may be useful, not only to identify new pockets and new hits, but also to support lead optimisation, providing new options for development.

Although blind docking is a quick and efficient method to identify initial binding sites, subsequent targeted docking at specific sites allows a more thorough and comprehensive search. In addition, analysis of binding sites from the blind docking output is manually intensive as Autodock does not assign compounds to each binding site.

For PTP1B we selected the two highest density clusters from the initial blind docking (1 and 4 in Figure 2) to perform targeted docking investigations with VSpipe Vina (see methods for details). Docking was done with the ChemBridge diverset library (50,000 compounds) and results were filtered by ΔG to select the top 500 compounds for each site. Cluster 1 binders ranged between −8.9 and −7.7 kcal/mol, and cluster 4 binders ranged between −11.6 and −9.4 kcal/mol.

#### 2.5.2. Analysis of Targeted Docking at the Active and Allosteric Sites

Selection of compounds for further experimental validation could be done on the basis of the ΔG scoring alone provided in the summary file. However, it is also useful to analyse other properties that impact on efficiency and druggability and to use them for cluster comparison. VSpipe generates a number of plots to assist in this selection using the filtering script (Section 2.2 and methods). Analysis of the top 500 compounds obtained for clusters 1 and 4 was done using the generated plots (Supplementary Figures S4 and S5). For example, PSA plots showed that the distribution range for the top compounds was similar for both clusters: 0–100 Å_2_. However, the percentage of compounds with PSA less than 50 Å_2_ was considerably different, with only 13% at cluster 1 compared to 34% at cluster 4. This indicates that cluster 1 compounds are more polar, which is a common trait in PTP active site inhibitors [3,4]. The analysis of other chemical properties is also available through the MW, logP and HBA plots (Supplementary Figures S4 and S5).

In addition, VSpipe generates LEI plots, which combine the chemical and biological space, providing information not only on the potency of the inhibitors, but also on their drug-like properties [12,32]. For PTP1B, we found these plots to be better discriminators of the two pockets than the chemical properties alone.

**Figure 4:**
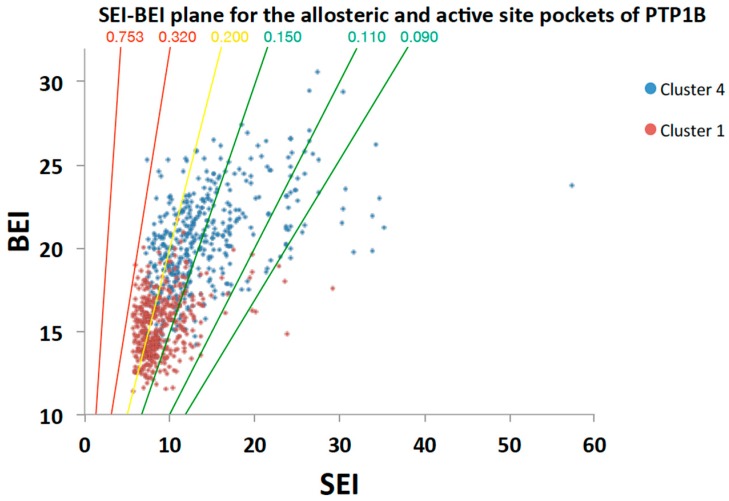
SEI-BEI plot for cluster 1 and 4. Selected ligands (top 500 ranked by ΔG) from the targeted docking are shown. Red dots represent ligands from the active site cluster 1 and blue dots represent ligands from the allosteric site cluster 4. The PSA/MW ratios (0.753, 0.320, 0.200, 0.150, 0.110, 0.090) are plotted as lines and coloured according to drug-like chemical space with 0.753–0.320 being a poor region (red), 0.200 acceptable (yellow), and 0.150–0.090 a good drug like space (green).

Of particular interest is the SEI-BEI plot (Figure 4), which describes the relationships between molecular weight, polarity, and the estimated potency of the compounds. The PSA/MW ratio planes (0.753, 0.320, 0.200, 0.150, 0.110, 0.090) were included on the SEI/BEI plot as they are a useful reference to classify the chemical-biological space according to drug-like properties of the compounds [13]. This way we defined 0.753–0.320 as a poor region, 0.200 as acceptable, and 0.150–0.090 as a good drug-like space (Figure 4). Distribution of the top 500 compounds for the allosteric site (cluster 4) is wide and shifted towards the favourable PSA/MW gradients 0.200–0.090. In contrast, the distribution of the top 500 active site compounds (cluster 1) is narrower and centred mostly around the 0.2 PSA/MW gradient (Figure 4). This restricted chemical space is typical of PTP active site inhibitors [13], where increases in potency are usually associated with increases in polarity, thus shifting to the unfavourable chemical space (top left Figure 4). Therefore, cluster 4 offers a greater number of starting points for drug discovery in a more drug-like space than cluster 1. Obviously, the range of BEI and SEI values will vary depending on the target and the compound library used. However, the interpretation of the SEI/BEI and compound selection criteria will remain the same.

**Figure 5:**
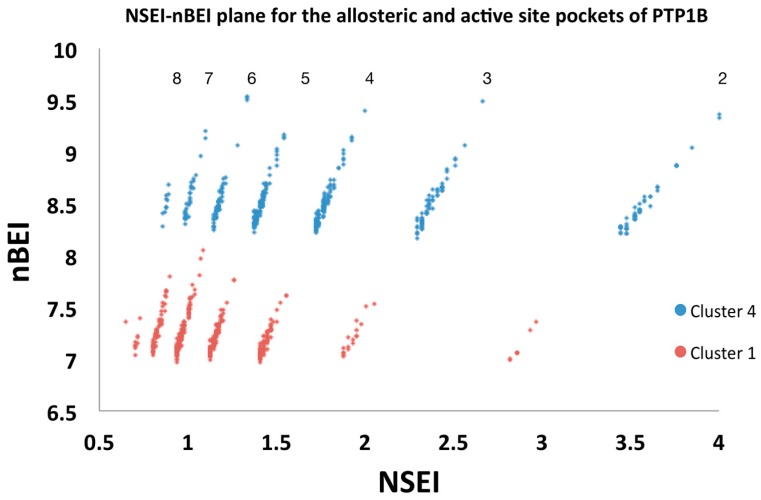
NSEI-nBEI plot for cluster 1 and 4. Selected ligands (top 500 ranked by ΔG) from the targeted docking are shown. Red dots represent ligands from the active site cluster 1 and blue dots represent ligands from the allosteric site cluster 4. Each diagonal line of compounds represents a specific NPOL (number of N and O atoms in the compound) as given by the number above each diagonal line.

VSpipe also generates NSEI/nBEI plots (Figure 5), in which compounds are sorted according to their number of polar atoms, nitrogen and oxygen (NPOL). Each NPOL plane contains compounds with the same number of polar atoms (2, 3, 4, 5 … etc.). Polarity increases counter-clockwise by NPOL, whilst ligand efficiency increases along each NPOL plane [12,13].

In our case study, the distribution of binders is shifted upwards and to the right for cluster 4 with respect to cluster 1. Thus, allosteric binders on the same NPOL plane represent higher efficiency ligands than those binders at the active site.

The population distribution of the top 500 compounds for cluster 1 is mainly on the NPOL 5 to 7 planes (with best scorers located on NPOL 6), whereas for cluster 4 the NPOL 3 to 5 lines are most populated, together with a considerably larger population on the NPOL 2 (with best scorers located on NPOL 2, 3, 4 and 6) (Figure 5). This indicates that the allosteric site is more tolerable of varying polarity. In contrast, the chemical space for the active site binders is more limited and confirms how critical polarity is for binding at this site.

In summary, the LEI plots clearly emphasise the differences between the two clusters of compounds, with cluster 4 residing in a more druggable space. Thus, comparison of the LEI profiles generated by VSpipe for different clusters is a useful approach to guide compound selection during the early stages of drug discovery. This approach may result in the progression of more diverse compounds than would have been achieved if potency (ΔG) alone is the driving factor. Furthermore, it can help in deciding on the mechanism of action to be exploited, in this case allosteric inhibition versus active site inhibition.

## 3. Discussion

We have developed VSpipe, a fast, efficient, and user-friendly pipeline to perform structure-based VS in a local or cluster mode. The main advantage of VSpipe is the concatenation of different tools with the corresponding input and output files. This includes tasks such as creating, removing, formatting, moving, and renaming files and directories, setting absolute and relative paths, executing different tools in a specific order and adding new information to existing files. VSpipe reduces the time the users have to spend converting or modifying files from one tool to another. This minimises human error, making it more efficient to manage files and directories and easier for the users to pre-define the options needed to run the VS. Specifically, it concatenates the two main modules of a VS: (i) the preparation of suitable atomic coordinates for the protein receptor and ligands; and (ii) the molecular docking. Importantly, VSpipe offers a filtering feature to assist in the assessment and selection of results. VSpipe is flexible, allowing users to take decisions at each step in the process, like the choice of which compound library to use or docking strategy, and whether a filtering step is required.

In addition to the summary output files with the physicochemical properties, VSpipe generates graphical plots calculated from these data. The information in the plots can be readily interpreted and utilised to guide the selection of suitable starting chemical points for drug discovery. Of particular interest are the LEI plots, which help in the evaluation of the drug-like properties of compounds. The LEI plots also allow comparison between related targets to explore specificity (i.e., human versus pathogen homologues), or between different binding sites within the same target. Likewise, comparison of the LEI profiles of different libraries against the same target may also provide useful guidance in the selection of commercially available compounds to build sufficient diversity for experimental evaluation.

We have demonstrated in our case study the value of using blind docking to identify ligand binding sites on PTP1B. We also demonstrated that a small fragment library is sufficient to identify functionally relevant sites as well as new binding sites. Blind docking has been previously used to successfully identify functional pockets on other targets [33], but this is the first report of its application to PTPs. Subsequent targeted docking around chosen pockets, allowed the assessment of the drug-like properties of binders at different sites, concluding that targeting the active site of PTP1B may not be conducive to inhibitors with great drug-like properties, as previously reported [4,13]. Instead, exploiting allosteric binding offers a larger and more diverse drug-like space. This is a particularly important issue for PTP targets because of the charged nature of their active site. In fact, our results indicate that the allosteric site binders have better drug-like properties, matching reported cell activity for PTP1B inhibitors that bind at this site [27]. Our results also suggest new binding sub-pockets that could be exploited in further development of allosteric inhibitors of PTP1B.

The use of VSpipe blind docking together with the analysis of ligand efficiency indices can be applied to any PTP and more widely to any target for which the structure is available (experimentally or by homology modelling), aiding in the selection of initial hit compounds for experimental validation. We have also shown that our approach may be a useful tool to guide lead optimisation by identifying alternative pharmacophores or sub-pockets. Furthermore, the use of LEI plots generated in VSpipe may be a useful guidepost to facilitate the discovery of new PTP inhibitors or new mechanisms of inhibition allowing this enzyme class to reach its full potential as targets for therapeutic intervention.

## 4. Materials and Methods

### 4.1. Scripting Languages and Working Environment

#### 4.1.1. VSpipe-Local Mode

This semi-automated pipeline is a bash script that concatenates the usage of AutoDock, OpenBabel tools, and in-house Python scripts to carry out the VS. The user creates first a home directory from which VSpipe is executed. After that, the pipeline creates all the additional sub-directories that are populated during the VS (see Section 2.2, Figure 1, and Supplementary Materials Figure S1). The users also provide the absolute path to the AutoDock scripts according to their local installation. Since various versions and package sources are available, we decided that it was simpler to let the user define the local path for the executables and let the pipeline set the environment accordingly. The docking tools AD4 v4.2.6 and Vina v1.1.2 implemented in VSpipe are freely available and simple to install [34,35].

#### 4.1.2. VSpipe-Cluster Mode

The cluster mode carries out the same tasks as the local mode. However, the different steps have been split into more than one bash script in order to adapt them to run in a cluster mode. VSpipe-cluster has been designed and implemented on the DPSF computing cluster of the UoM. The system is currently configured with 54 nodes, each with two 8-core Intel CPUs, varying RAM (256 to 512 GB) and it is running on CentOS Linux 7.2. Open Babel, Anaconda, MGLTools, AD4, and Vina [34,35] are required to run VSpipe, hence installed on the DPSF. Afterwards, there are two steps to follow. The first script (*pipeline_executable*) prepares the architecture of directories and files required to run VSpipe-cluster. This script performs three main tasks: (i) creates the different directories that will be needed during the VS; (ii) asks the users to upload the input files; and (iii) saves the users’ choices for each step of the VS, which are then read by the next bash scripts. After that, if the users have not prepared their ligands before, they have to submit to the cluster two files: first the *prep.txt* and then, depending on the docking tool to use, *dockAjd.txt* (targeted docking with AD4); *dockVblindjd.txt* (blind docking with Vina); or *dockVlocaljd.txt* (targeted docking with Vina). The second script will run as a job dependency (note the “jd” term at the end of the filename), which means that it will not start until *prep.txt* (input files preparation) has finished. On the other hand, if the users have previously prepared their ligands, they do not need to submit the script *prep.txt*. They only need to perform the docking and submit one of the following scripts, depending on the docking approach they have chosen: *dockA.txt* (targeted docking with AD4), *dockVblind.txt* (blind docking with Vina), or *dockVlocal.txt* (targeted docking with Vina). These files are not job dependencies, so their file names do not contain the term “jd” to avoid confusion with the previous files discussed above. We emphasise that to run VSpipe-cluster in another HPC cluster, two issues should be considered: (i) ensure that all required dependencies are installed; and (ii) all commands to run the Vina-OpenMP and AD4-MPI are configured according to the cluster environment. Note that the latter might involve changes in the main code of the pipeline.

### 4.2. Testing the Parallelisation of AD4 and Vina

Both docking softwares, AD4 and Vina, were parallelised on the computing cluster in order to speed up the docking process of the VS when running large ligand libraries. We tested the performance of the OpenMP versions of AD4 and Vina and of the MPI version of AD4 and carried out a benchmarking, together with the performance of the VSpipe-local mode, assessing the computation time it took to run each of them.

### 4.3. Target Proteins and Ligands

VSpipe accepts structures from any receptor/target protein in PDB format, hence we recommend to use this format to ensure correct VSpipe performance. Regarding the ligands, VSpipe supports both individual ligand files and ligand libraries. The formats that VSpipe currently supports are: PDB, MOL, MOL2, SMI, CAN, and SDF. Currently, VSpipe uses 21 libraries of compounds and fragments (in SDF format) that are available from different commercial providers and that can be pre-formatted to be used by VSpipe: AnalytiCon Discovery, Asinex, ChemBridge, ENAMINE, InterBioScreen, Indofine Natural Products, Maybridge, Princeton Natural Products, Specs Natural Products, and Zenobia.

### 4.4. Filtering the Results After the VS

VSpipe includes a filtering step once the VS has finished. In total, the output files (*output.csv* and *output.tsv*) include 16 parameters with which the users can evaluate the physicochemical properties of the docked ligands: molecular weight (MW), cLogS, cLogP, hydrogen bond donors (HBD), hydrogen bond acceptors (HBA), PSA, rotatable bonds, ΔG, Ki, ligand efficiency, BEI, SEI, NSEI, NBEI, nBEI, and mBEI. To filter the ligands by property and threshold value, the users run the script *filtering.py* in order to filter the results according to their decision. This script creates a new directory with the filtered results: (i) new output files (filtered_output.csv, filtered_output.tsv) including only the ligands that meet the filtering criteria specified by the users; (ii) a subdirectory with the PDB ligands files that meet the filtering requirements; (iii) and the ligand efficiency plots of the filtered ligands. These plots are (a) HBA vs. number of compounds; (b) log P vs. number of compounds; (c) MW vs. number of compounds; (d) NSEI (−log_10_Ki/NPOL) vs. nBEI (−log_10_[(Ki/NHEA)]); (e) PSA-number of compounds; and (f) SEI (p(Ki)/(PSA/100 Å^2^) vs. BEI (p(Ki)/MW(kDa)).

### 4.5. Docking Parameters for PTP1B

For our analysis of PTP1B we used PDB ID:1T4J with the following grid centre for the blind studies: x = 56.500, y = 31.333, z = 22.113. The grid spacing was set to 0.375 Å. The box size was predetermined to be used with VSpipe when using Vina for blind docking, and it is big enough to include the whole protein surface during docking. We used various libraries including Asinex_BB_v123_SD, Maybridge_Pre_Fragment_NCO, IBScreen Natural Product, and the Chembridge Chem-diverset. For the targeted docking, the grid centre used for the active site was: x = 56.500, y = 31.333, z = 22.113 and, for the allosteric site, it was: x = 45.361, y = 16.583, z = 2.583. The size of the box for both targeted dockings was 15 × 15 × 15 Å_3_, and a spacing of 0.375 Å was used. Docking was done with the Chem-diverset library composed of 50,000 compounds.

## Figures and Tables

**Table 1:** VSpipe performance using AD4 or Vina. A comparison of the CPU time using VSpipe-local or VSpipe-cluster and the different docking methods (AD4 or Vina). The blind docking was carried out with the library Maybridge Pre-Fragment-NCO (98 fragments) on the target protein PTP1B.

Software	VSpipe Version	Docking Time
		(hh:mm)
AD4	Local PC	14:10
AD4 (not parallelised)	Cluster DPSF	06:37
AD4-OpenMP (8 cores)	Cluster DPSF	02:04
AD4-OpenMP (16 cores)	Cluster DPSF	00:45
AD4-MPI (60 cores)	Cluster DPSF	00:09
Vina	Local PC	00:48
Vina-OpenMP (8 cores)	Cluster DPSF	00:21
Vina-OpenMP (16 cores)	Cluster DPSF	00:13

## Supplementary Materials

The supplementary materials are available online.
