# Supplementary material for: VSpipe, an Integrated Resource for Virtual Screening and Hit Selection: Applications to Protein Tyrosine Phospahatase Inhibition
Source: Molecules. 2018 Feb 7;23(2):353. doi: 10.3390/molecules23020353 (PMC6017540; doi:10.3390/molecules23020353)
Supplement: Supplementary file 1 [file molecules-23-00353-s001.pdf]

Supplementary Material

VSpipe, an Integrated Resource for Virtual Screening and Hit Selection: Applications to Protein Tyrosine Phospahatase Inhibition

Sandra Álvarez-Carretero [1,2], Niki Pavlopoulou [1,3], James Adams [1], Jane Gilsenan [1] and Lydia Tabernero [1,*]

[1] School of Biological Sciences, Faculty of Biology Medicine and Health, University of Manchester, Manchester Academic Health Science Centre, Manchester M13 9PT, UK; sandra.ac93@gmail.com (S.á-C.); nikipavlopoulou@gmail.com (N.P.); james.adams-6@postgrad.manchester.ac.uk (J.A.); Jane.Gilsenan@manchester.ac.uk (J.G.)

[2] Current Address: School of Biological and Chemical Sciences, Queen Mary University of London, London E1 4NS, UK

[3] Current Address: Insight Centre for Data Analytics, NUIG, Galway, Ireland

[*] Correspondence: lydia.tabernero@manchester.ac.uk; Tel.: +44-161-275-7794

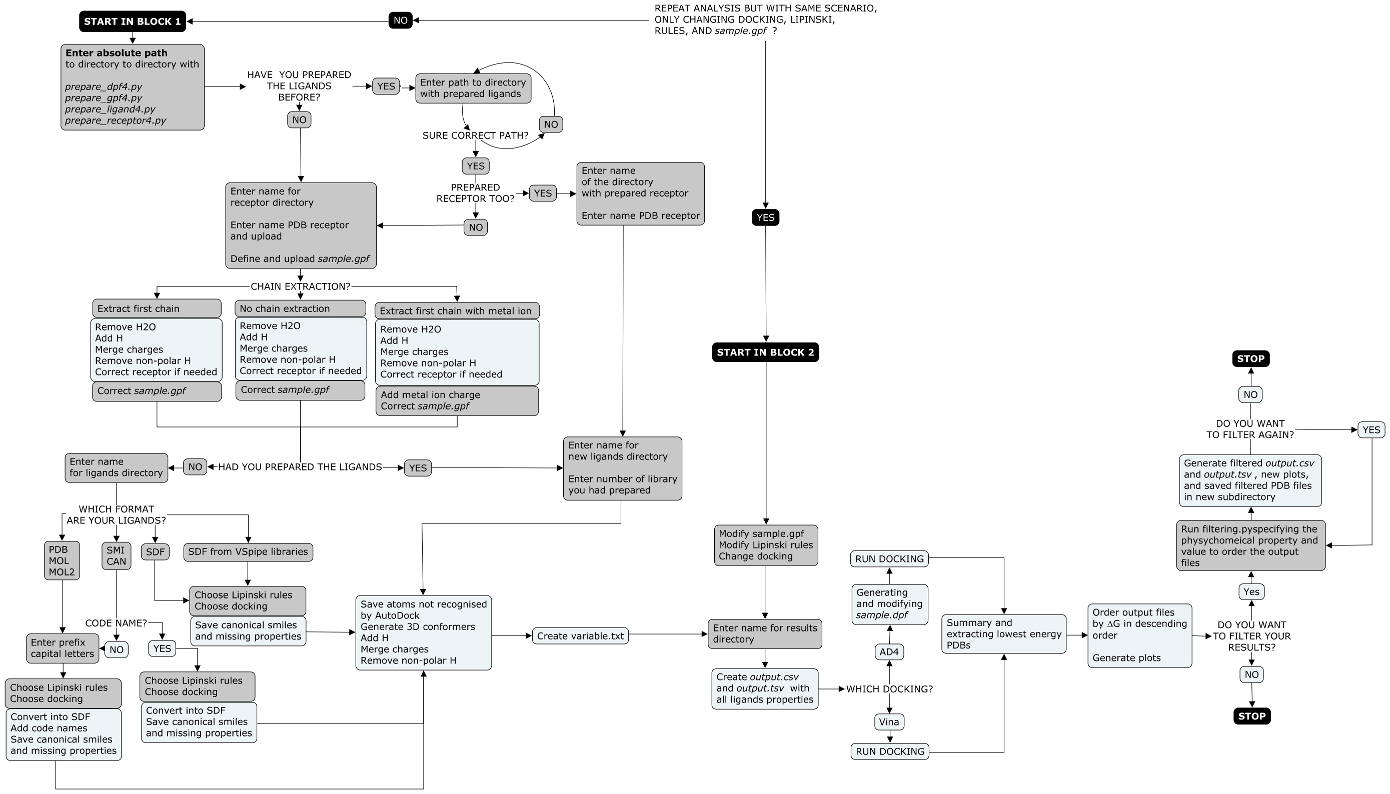

Figure S1. Workflow of VSpipe-local. This workflow diagram shows all possible user choices from the preparation of input files to the output summary of results. All user choices are saved as global variables and then written in the *variables.txt* file. The black boxes show the start and the end of each block and of VSpipe, the grey boxes show the information that users enter in the terminal or the tasks manually performed according to the instructions by VSpipe. Blue boxes show the tasks VSpipe performs after reading the information entered by the users.

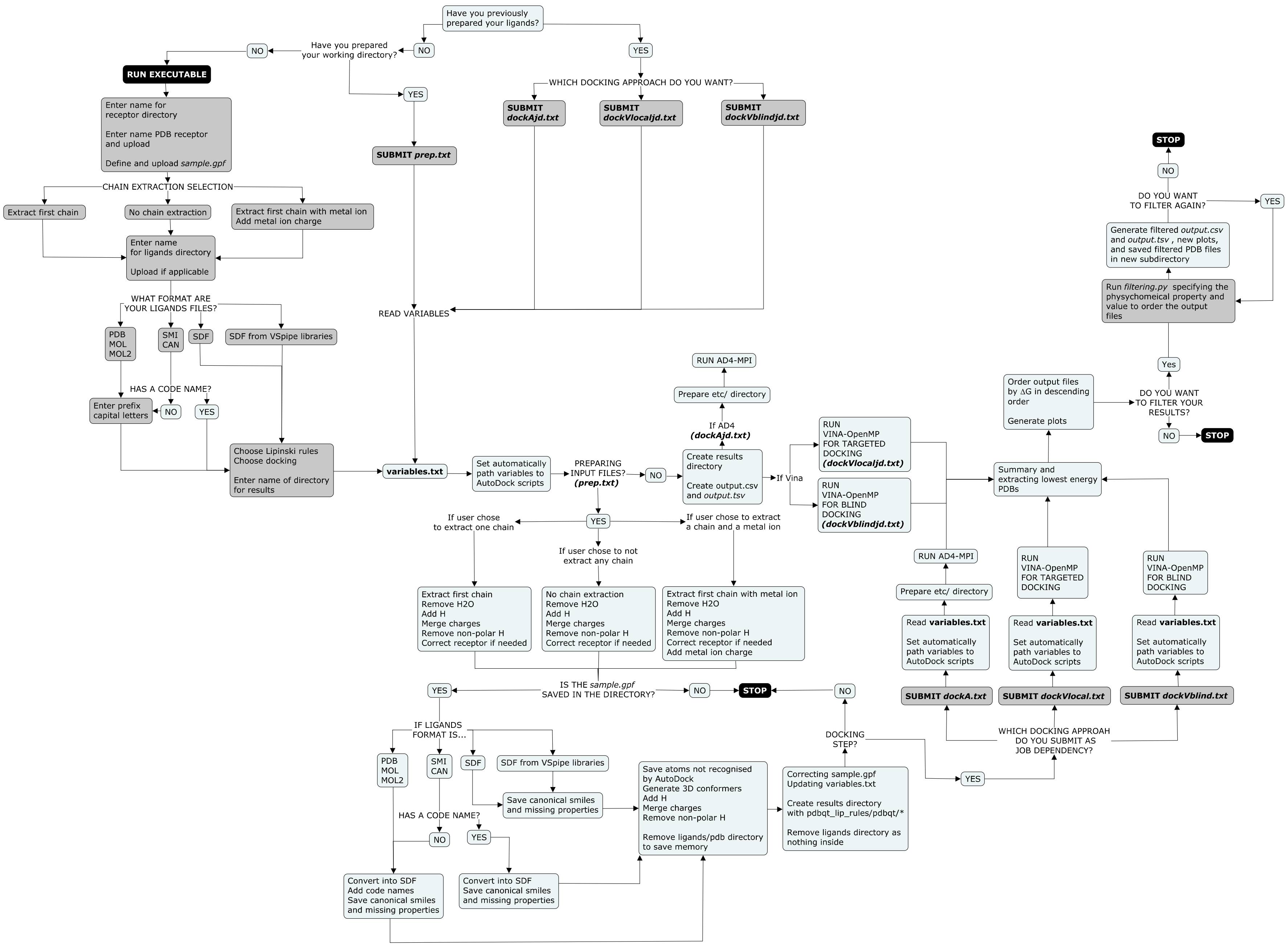

Have you previously prepared your ligands?
NO
YES
Have you prepared your working directory?
NO
YES
RUN EXECUTABLE
Enter name for receptor directory
Enter name PDB receptor and upload
Define and upload sample.gpf
CHAIN EXTRACTION SELECTION
Extract first chain
No chain extraction
Extract first chain with metal ion Add metal ion charge
Enter name for ligands directory
Upload if applicable
WHAT FORMAT ARE YOUR LIGANDS FILES?
PDB MOL MOL2
SMI CAN
SDF
SDF from VSpipe libraries
HAS A CODE NAME?
Enter prefix capital letters
NO
YES
Choose Lipinski rules Choose docking
Enter name of directory for results
SUBMIT prep.txt
WHICH DOCKING APPROACH DO YOU WANT?
SUBMIT dockAjd.txt
SUBMIT dockVlocaljd.txt
SUBMIT dockVblindjd.txt
READ VARIABLES
variables.txt
Set automatically path variables to AutoDock scripts
PREPARING INPUT FILES? (prep.txt)
NO
YES
Create results directory
Create output.csv and output.tsv
If AD4 (dockAjd.txt)
Prepare etc/ directory
RUN AD4-MPI
If Vina
RUN VINA-OpenMP FOR TARGETED DOCKING (dockVlocaljd.txt)
RUN VINA-OpenMP FOR BLIND DOCKING (dockVblindjd.txt)
If user chose to extract one chain
If user chose to not extract any chain
If user chose to extract a chain and a metal ion
Extract first chain Remove H2O Add H Merge charges Remove non-polar H Correct receptor if needed
No chain extraction Remove H2O Add H Merge charges Remove non-polar H Correct receptor if needed
Extract first chain with metal ion Remove H2O Add H Merge charges Remove non-polar H Correct receptor if needed Add metal ion charge
IS THE sample.gpf SAVED IN THE DIRECTORY?
YES
NO
STOP
IF LIGANDS FORMAT IS...
PDB MOL MOL2
SMI CAN
SDF
SDF from VSpipe libraries
Save canonical smiles and missing properties
HAS A CODE NAME?
NO
YES
Convert into SDF Add code names Save canonical smiles and missing properties
Convert into SDF Save canonical smiles and missing properties
Save atoms not recognised by AutoDock Generate 3D conformers Add H Merge charges Remove non-polar H
Remove ligands/pdb directory to save memory
Correcting sample.gpf Updating variables.txt
Create results directory with pdbqt_lip_rules/pdbqt/*
Remove ligands directory as nothing inside
DOCKING STEP?
NO
YES
WHICH DOCKING APPROAH DO YOU SUBMIT AS JOB DEPENDENCY?
SUBMIT dockA.txt
SUBMIT dockVlocal.txt
SUBMIT dockVblind.txt
Read variables.txt
Set automatically path variables to AutoDock scripts
Prepare etc/ directory
RUN AD4-MPI
RUN VINA-OpenMP FOR TARGETED DOCKING
RUN VINA-OpenMP FOR BLIND DOCKING
Summary and extracting lowest energy PDBs
Order output files by ΔG in descending order
Generate plots
DO YOU WANT TO FILTER YOUR RESULTS?
NO
STOP
Yes
Run filtering.py specifying the physychomeical property and value to order the output files
Generate filtered output.csv and output.tsv , new plots, and saved filtered PDB files in new subdirectory
DO YOU WANT TO FILTER AGAIN?
YES
NO
STOP

Figure S2. Workflow of VSpipe-cluster. The diagram includes the different bash scripts the users can submit in the cluster mode. If the users have not run any previous VS before, VSpipe starts with the executable file to prepare the working directory. Once the input files are in the directories created by this script, the users submit the script *prep.txt* to the cluster to prepare the receptor and the ligands. Afterwards, a job dependency script is used to perform the docking. The users have to decide which docking approach to use submitting either *dockADjd.txt*, *dockVblindjd.txt* or *dockVlocaljd.txt*. If users have previously prepared the ligands VSpipe skips the preparation step and goes directly to the docking. In this instance, there are no job dependencies as there is no other script to run before. The *dockAD.txt*, *dockVblind.txt*, and the *dockVlocal.txt* scripts perform the docking in the same way than the other scripts submitted as job dependencies. The black boxes show the start or the end of VSpipe, the grey boxes show the tasks that they have to be manually performed according to the instructions, and the blue boxes show the tasks VSpipe performs after reading the information entered by the users.

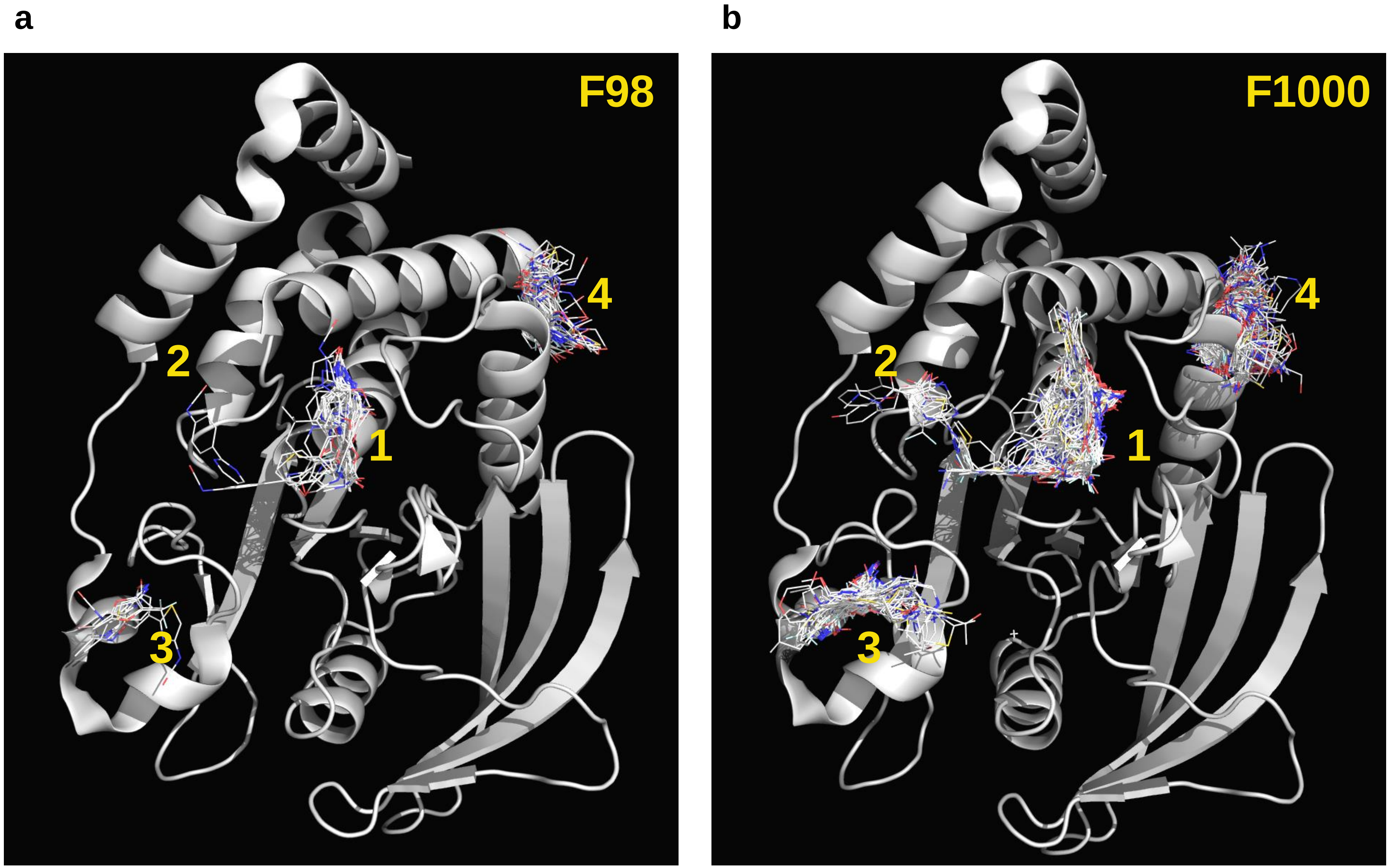

Figure S3. Cluster distribution from VS with fragment libraries. Blind docking results from VSpipe using PTP1B (PDB ID: 1T4J) and the **a)** Maybrigde pre-fragment (NCO) library (F98) or **b)** the Maybridge R03 1000 fragment library (F1000). The structure of PTP1B is shown as a grey cartoon with compounds shown as lines. Clusters match sites of functional importance: 1) active site, 2) secondary pTyr site, 3) centred on L41 4) allosteric binding site.

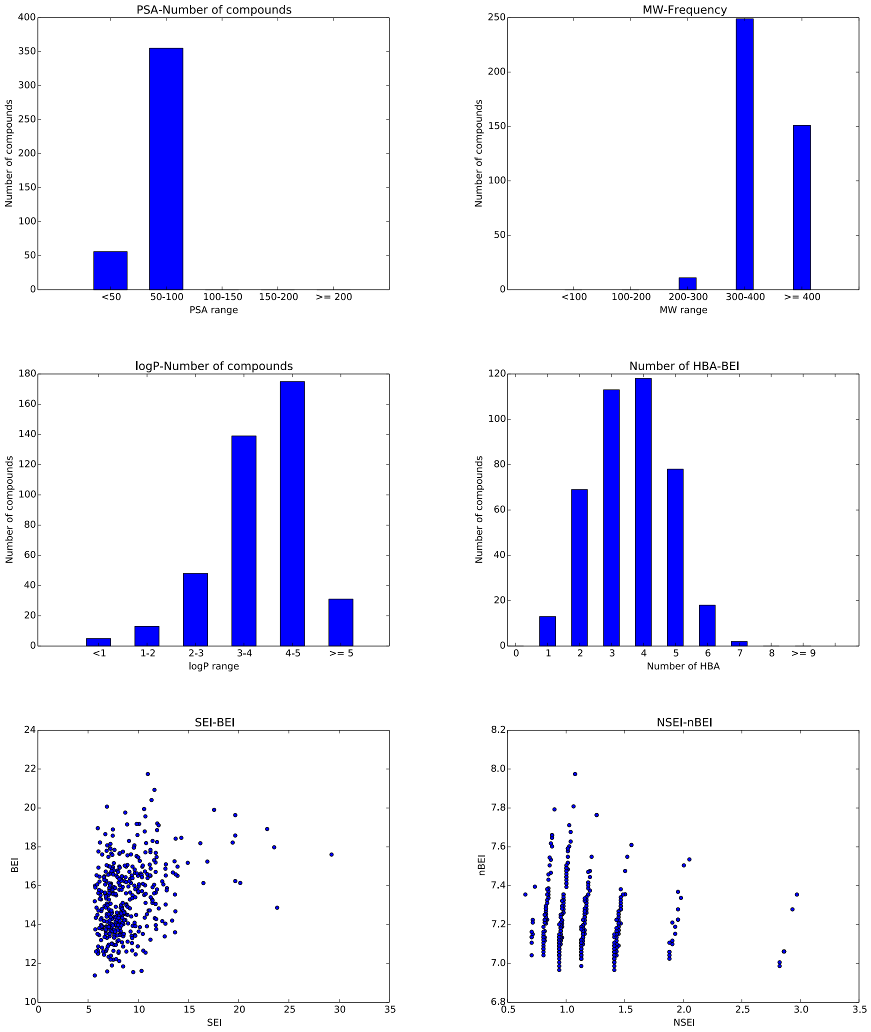

Figure S4. VSpipe plots for the targeted VS at the PTP1B active site. Plots produced by the filtering script for the top 500 binders at the active site. PSA plot, Mw plot, logP plot, HBA plot, SEI-BEI plot and NSEI-nBEI plot.

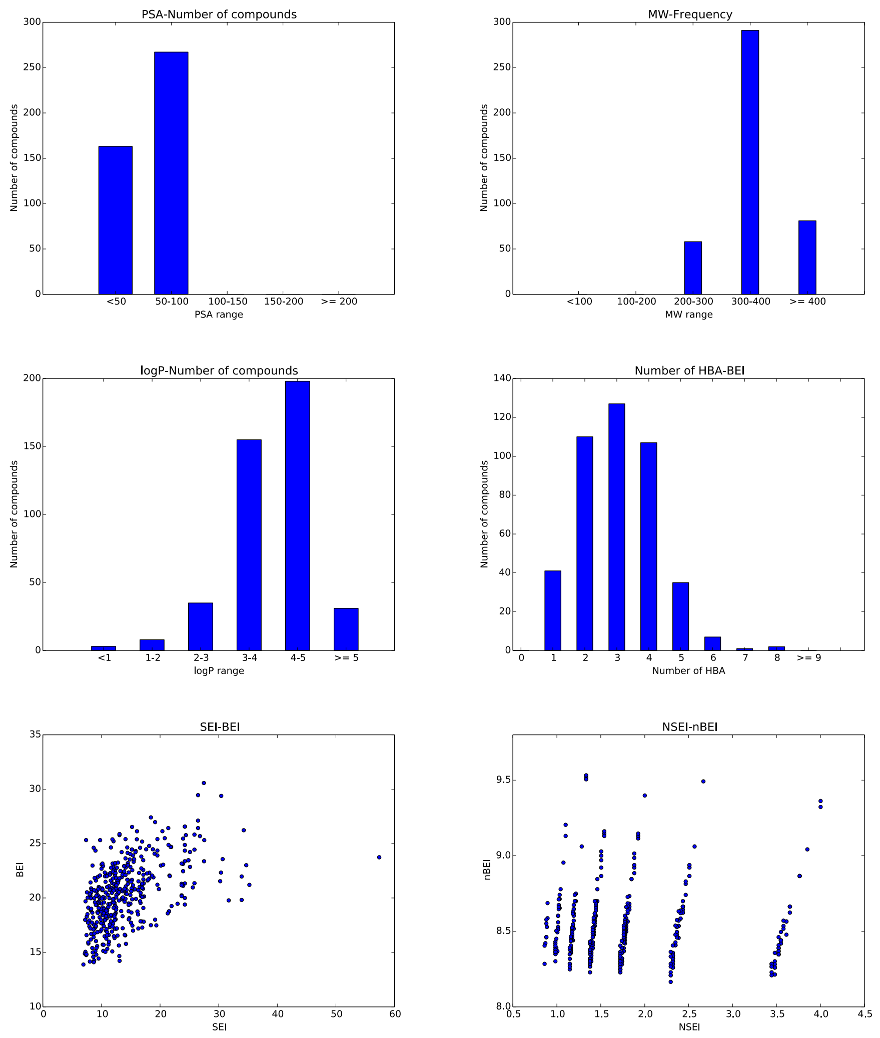

Figure S5. VSpipe plots for the targeted VS at the PTP1B allosteric site. Plots produced by the filtering script for the top 500 binders at the allosteric site. PSA plot, Mw plot, logP plot, HBA plot, SEI-BEI plot and NSEI-nBEI plot.
